# A realist evaluation of the continuum of HIV services for men who have sex with men

**DOI:** 10.1186/s12981-021-00396-2

**Published:** 2021-10-09

**Authors:** Willy Dunbar, Marie Colette Alcide Jean-Pierre, Jacky S. Pétion, Aline Labat, Nathalie Maulet, Yves Coppieters

**Affiliations:** 1 Health Systems and Policies-International Health, School of Public Health, Université Libre de Bruxelles (ULB), Campus Erasme - Bâtiment A, Route de Lennik 808 - CP591, 1070 Brussels, Belgium; 2 The Haitian Study Group for Kaposi’s Sarcoma and Opportunistic Infections (GHESKIO Centers), Port-au-Prince, Haiti; 3 Faculté des Sciences de la Santé (FSSA), Université Quisqueya (UniQ), Port-au-Prince, Haiti; 4 Organization of Optimistic Professionals for the New Haiti (OPORHA), Port-au-Prince, Haiti; 5 Institute of Health and Society, Université Catholique de Louvain (UClouvain), Brussels, Belgium

**Keywords:** HIV, Men who have Sex with Men, Stigma, Continuum, Realist evaluation

## Abstract

**Background:**

Men who have Sex with Men (MSM) represent the risk group that are disproportionately most affected by the human immunodeficiency virus (HIV) and continue to drop-off from the steps of the continuum of HIV services that have been adopted to overcome poor engagement and retention in care. This realist evaluation aimed at: (1) describing the evaluation carried out in Haiti aiming to ascertain why, how and under which circumstances MSM are linked and retained along the continuum, (2) assessing the outcomes of this approach and (3) exploring the motivators and facilitators for the HIV continuum of services through mechanisms and pathways.

**Methods:**

Guided by a realist approach, first, an initial program theory (IPT) was developed based on literature and frameworks review, participant observations and discussions with stakeholders. Then, the IPT was tested using a mixed method explanatory study: a quantitative phase to build the continuum from a cross-sectional analysis, and a qualitative phase to explore the motivators and facilitators related to proper linkages along the continuum. Finally, the IPT was refined by eliciting the mechanisms and pathways for outcomes improvement.

**Results:**

The results showed that the current service delivery model is suboptimal in identifying, engaging, linking and retaining MSM, resulting in loss to follow-up at every step of the continuum and failure to fully realize the health and prevention benefits of antiretroviral. However, the mechanisms through which linkages across the continuum can be improved are: self-acceptance, sense of community support and sense of comprehensive and tailored HIV services. These mechanisms are based on 10 different pathways: self-esteem, awareness and pride, perception of HIV risk, pcceptance and HIV status, addressing community stigma, strengthening of MSM organizations and community networks, societal acceptation and tolerance, stigma reduction training for healthcare providers, engagement of peers as educators and navigators and, adapted services delivery through drug dispensing points and mobile technology and financial assistance.

**Conclusions:**

The study findings show that engagement, adherence and retention to the continuum of HIV service for MSM are affected by a multi-layer of factors, thus highlighting the importance of taking a comprehensive approach to improve the program.

## Background

Men who have sex with men (MSM) represent the risk group that are disproportionately affected by the human immunodeficiency virus (HIV) compared to the general population [1]. Data suggest that the risk of HIV acquisition among MSM was 22 times higher than it was among all adult men in 2018 [2]. Despite progress to control the HIV among MSM, biological, behavioral, legal, socio-cultural factors continue to hamper the global response [3]. Around the world—even in countries where same-sex practices, relationships, and marriages are legal—discrimination and homophobia persist. In varying degrees, this can impact MSM’s ability and willingness to access high-quality health services and information [4]. In many settings, criminalization of consensual, adult same-sex behavior, stigma, discrimination and violence against MSM has created an environment which compromises people’s human rights and where they are less likely to access essential health and HIV services [5].

As the health and prevention benefits of antiretroviral therapy (ART) in the management of HIV are now well documented, the world is currently pushing for fast-track in driving the 95–95–95 targets: that by 2030, 95% of people living with HIV know their HIV status, 95% of people who know their status are receiving treatment and 95% of people on HIV treatment have a suppressed viral load [6]. Behavioral prevention programs, early diagnosis, prompt linkage to sustained care, retention in care, receipt of ART, and viral suppression constitute points along a comprehensive continuum of HIV services. The term continuum refers to this sequence of steps a person with HIV takes from diagnosis, through linkage to care, receiving treatment until viral load is suppressed to undetectable levels. Each step in the continuum is marked by an assessment of the number of people who have reached that stage [7, 8]. However, current service delivery models are less than optimal in referring, linking and retaining MSM, resulting in lost to follow-up (LTFU) in the continuum of care and failure to fully realize the benefits of ART [9, 10].

In Haiti, although the response to HIV has seen important advances and continued reduction in incidence and expansion of treatment access for those living with HIV, the epidemic in MSM remains severe and yet poorly studied [11]. This is clearly one of the defining challenges ahead in the effort to control the HIV in the country. The past years has seen notable progress with a declining HIV prevalence, and treatment outcomes. This success is tied to a strong foundation for HIV care that has contributed in reducing the national HIV prevalence from 6.2% in 1993 to 2.0% in 2018 [11, 12]. However, the HIV prevalence among MSM was 18.2% in 2017 making them the most underserved population in term of HIV prevention and care. MSM are not yet adequately represented in the literature on HIV and of particular concern is the lack of data on the continuum of HIV services in Haiti, which is necessary to measure national progress against UNAIDS 95–95–95 targets [6, 13]. In addition, research methodologies that account for the role that contextual factors play—instead of controlling them—have still been scarcely used to improve the HIV services uptake among MSM [14].

As various strategies have been adopted to overcome some of the challenges cited above, the Linkages Across the Continuum of HIV Services for Key Populations Affected by HIV (LINKAGES) Program has been developed. Evaluations to assess the level of implementation of this intervention targeted MSM along the continuum are needed. Most importantly, studies to highlight the mechanisms that trigger linkage and retention in care are important. This article aims to fill this knowledge gap by: (1) describing the evaluation carried out in Haiti aiming to ascertain why, how and under which circumstances MSM are linked and retained along the care continuum, (2) assessing the outcomes of this approach and (3) exploring the motivators and facilitators for the HIV continuum of services through mechanisms and pathways.

## Methods

### Study conceptual framework

In this study, we applied the realist evaluation approach to explore the linkages across the continuum of HIV services for MSM in Haiti. Realist evaluation, proven to be useful when exploring complex health systems interventions, asks ‘what works?’, and also ‘how or why does this work, for whom, in what circumstances?’ [15]. The nature of realist evaluation lies in its conceptualization of the context, the mechanism and the outcome of a complex intervention [16, 17]. It starts by eliciting an initial program theory (IPT) which is tested through research. The data analysis serves to refine the IPT while identifying the mechanisms that trigger linkages and retention [18, 19].

### Overview of the LINKAGES

**Fig. 1:**
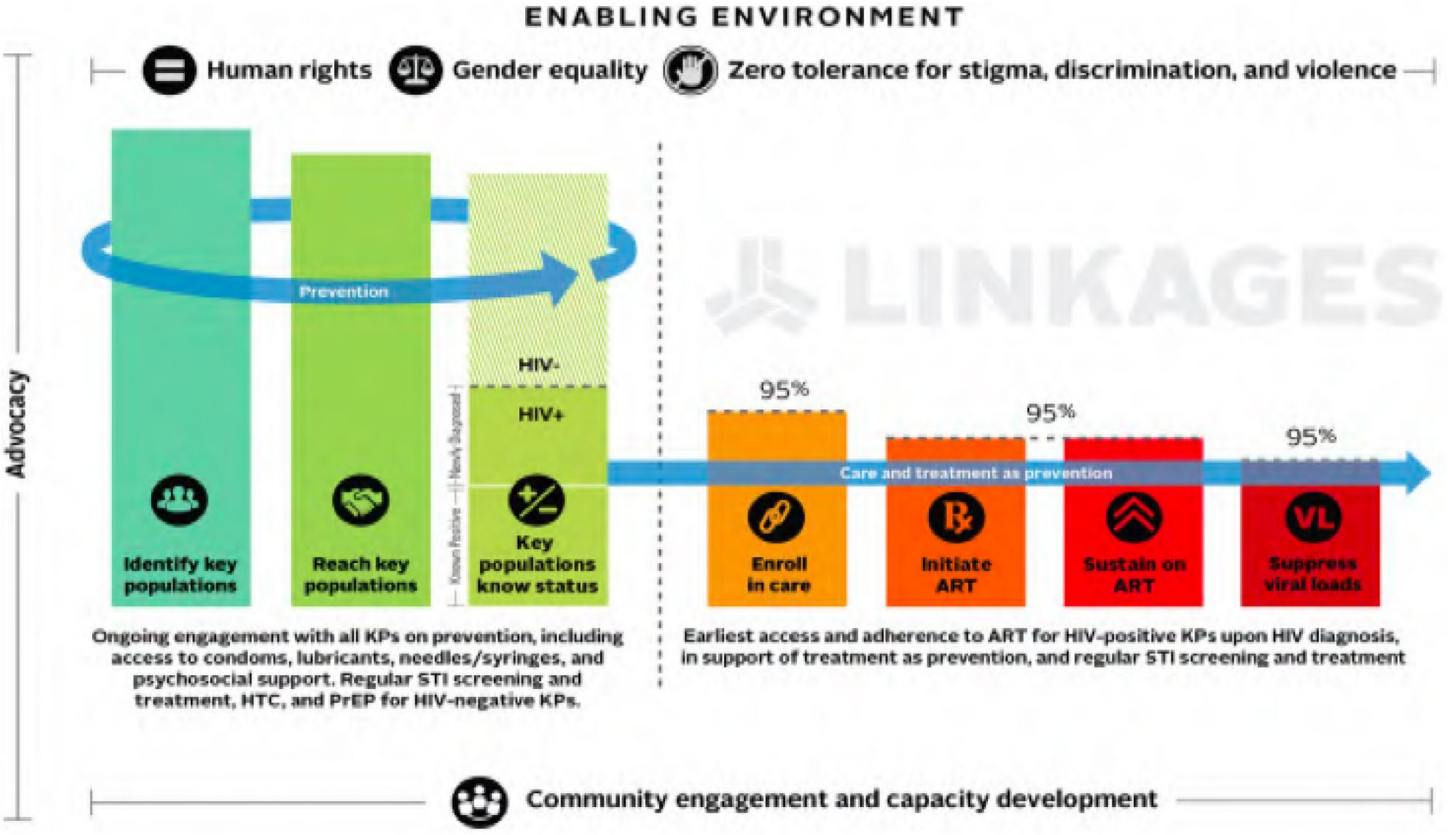
Cascade framework for HIV services along the continuum developed by Mills and Francis [37]. *KPs* key populations, *STI* sexually transmitted infections, *HTC* HIV testing and counseling, *PrEP* pre-exposure prophylaxis

The LINKAGES is a project that aims to accelerate the ability of governments, MSM organizations and private sector providers to collaboratively plan, deliver and optimize services that reduce HIV transmission among MSM and extend life for those who are HIV-positive. This intervention works with multiple stakeholders by: (1) identifying MSM and comprehensively assessing risk and service access, (2) diagnosing “leaks” within the HIV services cascade, (3) scaling up “what works” while innovating to ensure the most strategic use of resources and access to newly emerging technologies, (4) pulling down structural barriers and transforming MSM organizations, and (5) ensuring MSM interventions are sustainable [4]. The approach is summarized in the cascade framework that presents services along a continuum of HIV prevention, diagnosis, care, treatment, and viral suppression (Fig. 1). The cascade is aligned with the United Nations 95–95–95 targets.

We conducted the present study at the Groupe Haitien d’Etude du Sarcome de Kaposi et des Infections Opportunistes (GHESKIO) in Port-au-Prince, Haïti. GHESKIO works with the Haitian Government to implement the prevention and care model to a network of 27 healthcare centers throughout the country through training, monitoring and evaluation. This seamless integration of intervention components requires strong linkages among program elements so that HIV transmission is reduced and MSM diagnosed with HIV obtain early access to services. Thus, this model necessitates that MSM flow efficiently, consistently, and sustainably through the entire continuum.

### Overall study design, steps and phases

**Fig. 2:**
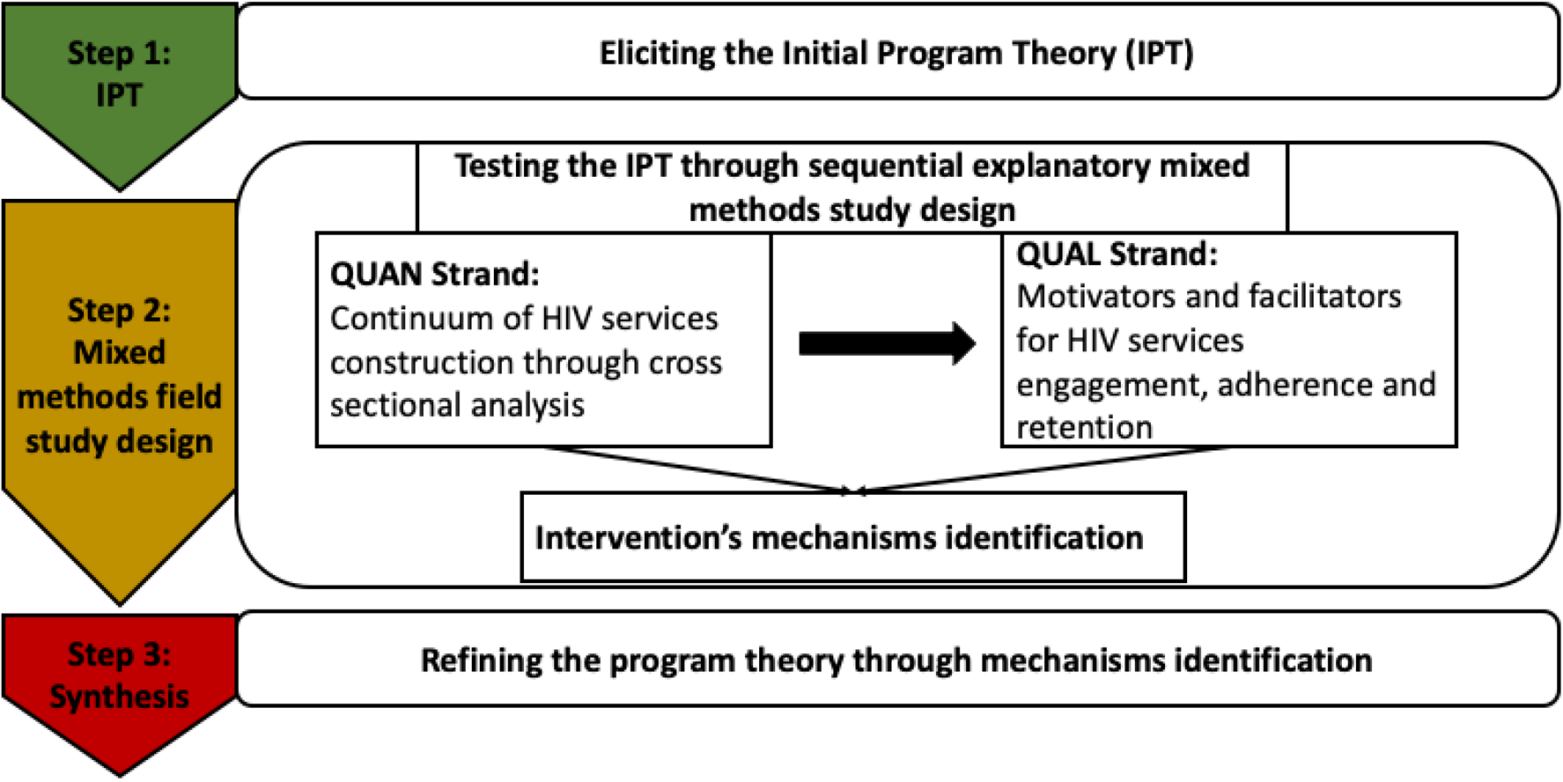
Study methodological process

The study methodology is divided in three steps: (1) initial program theory elicitation, (2) initial program theory testing through mixed method explanatory design and, (3) mechanisms identification and theory refinement (Fig. 2).

#### Step 1—Eliciting the IPT

This evaluation started with the formulation of the IPT. We explored the development of the continuum by reviewing program framework and reports, discussing with program coordinators, healthcare providers and MSM and participant observations at the health facilities. Guided by realist evaluation principles, the findings were analyzed thematically. This IPT served as a hypothesis that was tested through a mixed methods design.

#### Step 2—Testing the IPT

For this second step, we carried out a mixed methodology with a sequential explanatory design: a quantitative phase to build the continuum from a cross-sectional analysis, and a qualitative phase to explore the motivators and facilitators related to linkage along the continuum [20].

- Quantitative phase—Continuum construction and analysis:
  A cross-sectional analysis from programmatic and technical reports data of activities implemented from January 2017 to December 2018 were used to construct the continuum. Then, to build the cohort, a retrospective review of MSM living with HIV electronic medical records (EMR) was used to collect data. As an exploratory part in nature no formal sample size calculations were conducted. Patients aged 18 years and above and enrolled between January 2017 and December 2018 were screened for eligibility to be included in the study. Patient files were excluded if they missed data on important variables including date for start of care, sexual orientation and age. We calculated the proportion of participants who proceeded through specific steps in the cascade from prevention to viral suppression. In January 2020, we calculated the outcomes for the cohort overall. This period ensured follow-up 12 months after enrolment. The events of interests were the number of MSM reached through prevention and testing services, who tested positive for HIV, were linked with HIV care, initiated ART, were retained in care (had at least two outpatient visits at least 90 days apart after ART initiation), had a viral load test at around 6 months after ART initiation, achieved viral suppression (less than 200 copies/mL), and were active and on treatment or lost to follow-up (180 days without any visit to clinic after starting ART) as described by the program specific objectives.
- Qualitative phase—Generating mechanisms and exploring pathways for engagement, adherence and retention:
  We conducted 5 Key informants’ interviews to explore how the program works. Then, 27 in-depth interviews (12 with MSM who were LTFU and 15 with MSM who were active and on ART) were also conducted to study the perceptions of MSM regarding the HIV care continuum, their experiences and the potential motivators and facilitators for engagement, adherence and retention. The key informants involved five doctors, program coordinators and MSM peer educators. In order to ensure maximum variation, we managed to elicit views from MSM patients enrolled in care and receiving ART and from MSM who were LTFU. Those LTFU were contacted using the telephone contact extracted from the EMR. All interviews were conducted in French Creole using an interview guide and were recorded and transcribed verbatim. Saturation was reached after conducting 27 in-depth interviews. Our inductive approach to data analysis was based on the thematic analysis in line with the study aim [21, 22].

The methods were integrated through triangulation of quantitative analysis to assess the steps of the continuum and qualitative analysis to elicit mechanisms. While the retrospective cohort analysis provided an insight into the continuum of care and the outcomes of the MSM, the qualitative data analysis provided the evidence to strengthen every step in the cascade by identifying the mechanisms and pathways that can contribute to better outcomes.

#### Step 3—Refining the program theory through mechanisms identification

The aim of the third step was to identify mechanisms and pathways through an assessment of the continuum and a thematic content analysis. The IPT provided a basic framework on understanding how and why mechanisms generate the outcomes which are linkage to care, adherence and retention. Codes and themes were generated in regard to the context, mechanisms, pathways and intervention strategies in an iterative way.

### Trustworthiness of the work

Prior to data collection and in order to understand the contextual factors relating to the HIV care continuum for MSM, meetings and observations were held with MSM patients, and healthcare workers. Credibility was established by selecting key informants’ interviews and in-depth interviews to collect data while being familiar with the context. Dependability was established by describing the data analysis in detail and providing direct citations to reveal the basis from which the analysis was conducted. The citations used in this article were translated from French Creole into English with the help of a translator, to maintain accuracy and context as much as possible. Conformability and consistency of the analysis were established by holding meetings for the team to discuss preliminary findings, emerging codes and themes until consensus reached. To enhance the transferability of the findings, descriptions of contexts, selection of participants, data collection and analysis are provided in order to enable the readers the possibilities to determine whether the results of this study are transferable to another context [23, 24].

### Ethics statement

Authorization to conduct the study was obtained from the GHESKIO’s human right committee and the Cornell University Weil Medical College’s Research Ethics Board. At the level of the program, permission was obtained from the facility managers, and finally, consents from the participants and the key informants were obtained. Confidentiality was assured and data were anonymized.

## Results

### Step 1—Eliciting the IPT

**Fig. 3:**
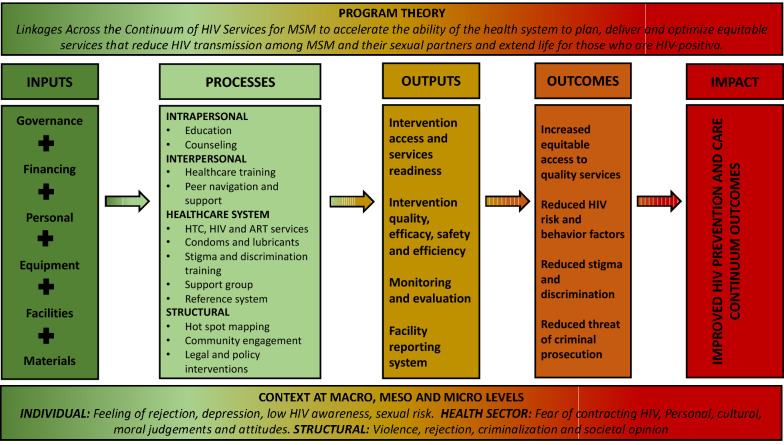
Initial program theory of how the linkages across the continuum of services improve HIV prevention and care outcomes for MSM. *HTC* HIV testing and counseling, *ART* antiretroviral

The IPT described how the implemented interventions within Haitian specific healthcare and structural contexts have produced the expected outcomes. This is the result from reviews of the grey literature, program frameworks and project combined with assumptions by stakeholders about what inputs and processes are required to ensure linkages across the continuum regarding the context [8, 25–27]. As presented in the Fig. 3, developing the IPT allowed us to identify the inputs (governance, financing, personal, equipment, facilities and materials) behind the intrapersonal, interpersonal, health system related and structural processes. The processes constantly drive the outputs (services access, readiness, quality, efficacy, safety, efficiency, monitoring, evaluation and reporting system) which resulted in the outcomes for increased equitable access to quality services, reduced HIV risk, behavior factors, stigma, discrimination, threat and criminal prosecution. The overall dynamic of the IPT is influenced by the macro, meso and micro levels of the context.

As one of the direct illustrations: input combining healthcare workers using adequate materials allow the conduct of community and institutional counselling and testing processes. These processes are directly linked to the outputs of quality, efficacy, safety and efficiency of the activities in order to produce a reduction of HIV risk and behavior factors among MSM as outcomes. These outcomes contribute to improved HIV prevention and care continuum outcomes.

### Step 2—Testing the IPT

#### The continuum of HIV services among MSM

**Fig. 4:**
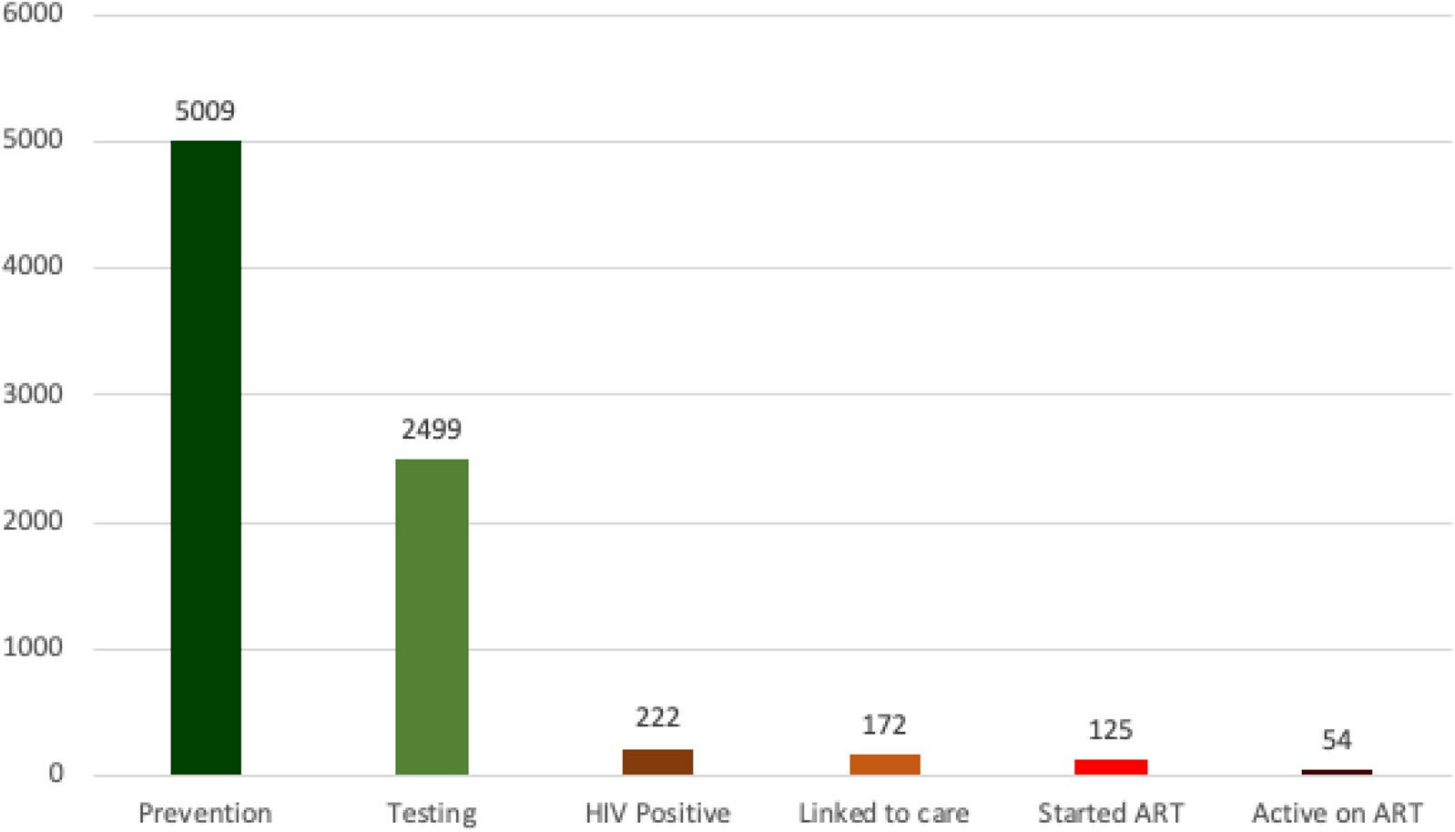
The continuum of HIV services for MSM

Linkages in the continuum are frequently inadequate at every stage of the HIV continuum of prevention, care, and treatment. Weak linkages among programs can be thought of as a leaky pipe along the continuum of HIV services. Outreach programs often refer MSM members to HIV testing and counseling (HTC), yet a large segment of those reached never actually go for an HIV test. If MSM members do obtain an HIV test, those who are HIV negative are only test once or infrequently, despite ongoing risk. Those diagnosed HIV positive leave the testing site without a referral to care and treatment. The journey of the MSM through the HIV continuum of services is depicted in Fig. 4. Between January 1st, 2017, and December 31, 2018. 5009 MSM were reached for prevention services at the community level. Of those reached, 2499 (49.9%, 95% CI 48.5–51.3) were tested for HIV, 222 (8.9%, 95% CI 7.8–10.0) had a positive test result for HIV. Of these, only 172 (77.5%, 95% CI 71.4–82.8) were linked to HIV care and 125 (72.7%, 95% CI 65.4–79.2) started ART and had a documented viral load test result. After 1 year of follow-up, among the 125 participants who started ART: 54 (44.6 95% CI 24.5–38.9) were active and on care, 59 (44.8%, 95% CI 27.2–41.9) were lost to follow-up and 8 (4.6% CI 20.3–89.5) were transferred out. In term of virologic profile, among the 125 who started ART, 98 (78.4%, 95% CI 49.2–64.5) achieved a suppressed viral load.

The demographic characteristics of the MSM who started ART are presented in Table 1. Most of them were in their 20 s (n = 66, 52.8%) with secondary and superior education level n = 95, 76%). Nearly half of them were unemployed (n = 47, 37.6%) and with an income of less than $5.000 US/year (n = 62, 49.6%). Most participants reported having sexual partners (n = 121, 97.6%), however, only a few (n = 8, 6.7%) disclosed their HIV status to their sexual partners and used condom on a regular basis (n = 16, 13.3%). Most of the participants reported being married to women (n = 100, 80%).

**Table 1:** Baseline characteristics of the 125 MSM living with HIV who started ART

Characteristics of the MSM	N (%)
Total	125
Age group	
18 to 29 yrs	66 (52.8)
30+ yrs	59 (47.2)
Education level	
None/primary	30 (24)
Secondary/superior	95 (76)
Occupation	
Unemployed	47 (37.6)
Occasional workers	42 (33.6)
Stable workers	36 (28.8)
Income	
No income	60 (48)
< $5.000 US/year	62 (49.6)
> $5.000 US/year	3 (2.4)
Sexual partner	
Yes	121 (97.6)
No	3 (2.4)
Disclosure of HIV status to sexual partner	
Yes	8 (6.7)
No	111 (97.3)
Condom use	
Always	16 (13.3)
Never	6 (5)
Sometimes	98 (81.7)
History of commercial sex	
Yes	27 (27)
No	73 (73)
Ever been married to a woman	
Yes	100 (80)
No	25 (20)
Have children	
Yes	54 (43.2)
No	71 (56.8)

### Eliciting mechanisms throughout the continuum

The formulation of the IPT resulted in a classification of the processes behind the continuum in four components: intrapersonal, interpersonal, healthcare systems and structural. The thematic analysis of the qualitative phase was consistent with this classification. As the findings were grouped by themes, three mechanisms were emerged: (1) self-acceptance, (2) sense of community support and (3) sense of comprehensive and tailored HIV services. For each of them, the pathways for engagement, adherence and retention were identified. Thus, we present the three mechanisms with their respective pathways and supporting quotations to illustrate the findings.

#### Mechanism 1: Self-acceptance

The most common recurrent theme across the interviews was the interactions of perceived stigma and willingness to engage in HIV continuum of services. Thus, self-acceptance is based on hope and confidence to achieve a complete acceptation of sexual orientation, perceived HIV risk and HIV status and can be activated by a combination of three different pathways: (a) self-esteem, awareness and pride, (b) perception of HIV risk and, (c) acceptance and HIV status.

- Pathway 1—Self-esteem, awareness and pride: according to the majority of MSM interviewed, self-esteem, awareness and pride are prominent determinants for mental health and well-being. They pointed out that those factors are precursors that play a significant role on steps to accept their sexual orientation. Thus, psychosocial constructs are mandatory to improve their willingness for their overall health and therefore for HIV prevention and care engagement.
  *“…We can only achieve great things once we reach pride regarding our homosexuality. When I was hiding, I didn’t have the mental freedom to take care of myself, even after knowing my HIV status.”. [IDIMSM0015]*
- Pathway 2—Perception of HIV risk: even though they fear of unintended disclosure and anticipated stigma, participants recognized that basic knowledge of a higher potential and vulnerability for contracting HIV among MSM represents a key factor for taking part in HIV prevention and testing activities.
  *“…Doc, I didn’t know that I had to use condoms because I don’t have sex with women. After the training sessions, the trainer explained how we can get infected with HIV… He explained how it is easy for gays to be infected. At that particular moment, I realized that I’ve been at risk without knowing. This is why I am now a trainer, to help others understand that and participate in community meetings.” [IDIMSM0004]*
- Pathway 3—Acceptance and HIV status: although MSM reported a variety of feelings after their HIV diagnosis, they explained that being able to cope with their HIV status leads to self-acceptance, self-stigma mitigation and peer support seeking through disclosure.
  *“What could I do more? Nothing. I simply accept my result and I realized that I am also lucky to be able to receive treatment.” [IDIMSM0011]*

#### Mechanism 2: Sense of community support

Social relations among MSM represent a reliable source of better health outcomes. However, community stigma can impede the progress. Thus, increase social relation and empowerment lead to an established network essential to community education and awareness on issues linked to sexual orientation and HIV infection stigma and discrimination.

- Pathway 4—Addressing community stigma: according to the participants, community and family stigma have contributed to a large number of MSM refusing preventive care, missing their appointments in the clinic and discontinuing their treatment. Thus, community education and awareness on issues linked to sexual orientation and HIV infection play a major role in encouraging care-seeking behavior, adherence and retention.
  *“It is difficult to take the medications and come for appointments when everyone at your house and neighborhood don’t accept the fact you are gay.” [IDIMSM0014]*
- Pathway 5—Strengthening of MSM organizations and community networks: the key informants and the MSM provided insights about the role of organizations and networks in promoting social activities to improve confidence and allow free exchange of ideas, coordination of participative projects towards achieving effective results in engaging MSM in prevention and care.
  *“We are having important help from the MSM networks. They help us reach other MSM and we are always invited to conduct training and counseling during their weekly meetings. If someone miss appointments, they call him or visit him…During the meetings they tell their own stories to motivate others” [KIICHW0002]*
- Pathway 6—Societal acceptation and tolerance: MSM explained that highly stigmatized by both religious and social norms, their homosexual practices are driven underground. Besides, in some cases they face violence perpetuated at a community level. They advocated for better legal protections in order to promote tolerance which can decrease HIV vulnerability and increase access to sexual and HIV information, testing, prevention and care.
  *“If I don’t feel safe in the community, I will not go to the activities.” [IDIMSM0013]*
  *“I was afraid to go in the meetings at first, but my friend picked me up, we go together, this is the reason why I stay.” [IDIMSM0016]*

#### Mechanism 3: Sense of comprehensive and tailored HIV services

Sense of comprehensive and tailored HIV services represents a step towards trust and confidence in the health systems. It is a mechanism based on three different pathways: (1) stigma reduction training for healthcare providers, (2) engagement of peers as educators and navigators and, (3) adapted services delivery through drug dispensing points and mobile technology and (4) financial assistance.

- Pathway 7—Sexual stigma reduction training for healthcare providers: some participants reported having experienced stigma when they interact with health workers, while participating in community initiatives and clinical activities. Therefore, they mentioned needs to properly deliver sexual stigma reduction training as a key to successful link to care.
  *“I think the nurses also need training to know how to talk to gays. I don’t like the way they refer to me, or call me when I am waiting…” [IDIMSM0017]*
- Pathway 8—Engagement of peers as educators, navigators, and treatment supporters: MSM and key informants recognized the importance of peer educators and navigators as important members of the team for essential prevention, and care promoting strategies. They specifically expressed their involvement in subtle and comprehensive discussions to change risky sexual behaviors and to increase adherence to care.
  *“I felt difficult to go there (hospital) at first. But, with the peer educator, I understood the reasons why it is important for me to take the medications, go to the appointments and protect my friends.” [IDIMSM0012]*
- Pathway 9—Adapted services delivery through drug dispensing points and mobile technology: in order to avoid long waiting time at the clinic and work time conflicts and absence due to the country political instability, the solutions that MSM proposed is to have adapted services where they are able to receive their drugs in other clinical settings, local pharmacies and community locations with respect to confidentiality.
  *“During the appointment, I mentioned the fact that I am working. Now, I receive a message on my phone to remind me of the appointment… I can come on Saturday…Since then, I have had no more problems with my director.” [IDIMSM0008]*
- Pathway 10—Financial assistance: as lack of employment represented a key factor to inability to afford indirect expenses such as transportation to the clinic for routine visits, MSM advocated for economic support such as transportation fees, decrease of visit frequency and implementation of mobile clinics.
  *“I missed some visits because I didn’t have the money to go. But, since they started giving us the fees, it is so much easier for me to go.’ [IDIMSM0006]*

### Step 3—Refining the program theory through mechanisms identification

**Fig. 5:**
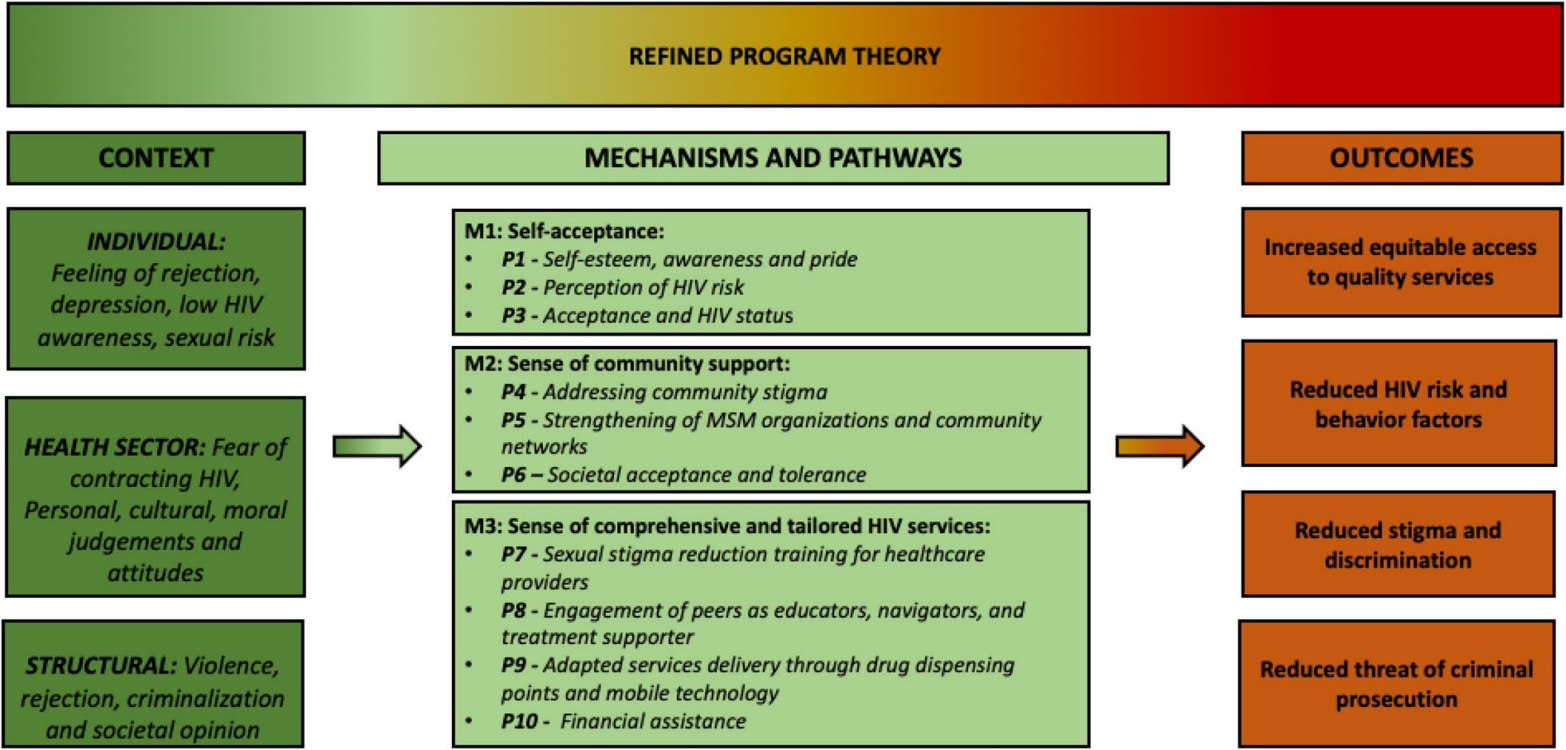
Refined program theory illustrating the mechanisms and pathways to engagement, adherence and retention throughout the continuum

The IPT provided a basic framework on understanding the limitations and barriers towards proper linkages and showed how and why mechanisms generate the outcomes. Therefore, we present an exploration of how, why, for whom, and in which circumstances particular mechanisms work. For each outcome, we tested the association with an identified mechanism and pathways taking into consideration the context. The CMO configuration was then refined during this process. Thus, Fig. 5 represent the refined program theory by using an interpretive approach to synthesize evidence to reveal how processes interact with context to trigger mechanisms in order to produce the outcomes.

## Discussion

Considering that programs are embedded theory, the use of realist method in this study allows us to conduct the evaluation while capturing the gaps between each step of the continuum, the contextual factors and a package of propositions for its improvement [28].

Since the beginning of the HIV epidemic, MSM have been one of the most at-risk groups [29]. Due to many contextual factors, and particularly because they are subjected to self, perceived stigma and discrimination, proper engagement in health services is difficult [30]. While many interventions have been tested for MSM, yet some struggle to accept and adjust to their HIV diagnosis, decided not to initiate ART and some are lost to follow-up [31]. This study revealed the pattern for MSM in Haiti but also tried to identify relevant mechanisms to address those issues. We used a mixed-approach surrounded by a theory-based method to build the continuum and gain insights into motivators and facilitators to fill the gaps. Through quantitative assessment we build the continuum, identify the breaches, and qualitative exploration, we identified the pathways and mechanisms to achieve key milestones for overall improvement.

Our main findings proved that the current service delivery models are less than optimal in engaging and retaining MSM, resulting in lost to follow-up and failure to fully realize the health and prevention benefits of ART. However, we also explored valuable insights in identifying pathways lead to better engagement, adherence and retention through mechanisms identification. Our results demonstrate that the 95–95–95: the fast-track is far to be reached as MSM are lost from the prevention step. These findings were in accordance with other studies evaluating the continuum in many different contexts [32–34]. However, some important considerations must be highlighted about these results: first, attracting MSM through community activities is difficult in a country where homosexuality is still largely disapproved; second, disclosure of sexual orientation during consultation is suboptimal; therefore, our data of MSM in care only represents patients who voluntarily disclosed their sexual orientation. In addition, viral suppression appears relatively acceptable among those who started ART even though many of them were already lost to follow-up.

We found that disclosure was rarely practiced by MSM. Only 6.7% reported disclosing their HIV-positive status to their partners while 98% don’t use condom on a regular basis. Given the critical role of unprotected anal sex in HIV transmission, the low reported disclosure rate is concerning [35]. Besides, 80% have already been married to a woman. Although some MSM may wish to engage in relationships with both men and women, heteronormative culture may lead MSM to marry women regardless of their preferences, concealing their same-sex behavior and placing their wives at risk of HIV. This gendered vulnerabilities that contribute to HIV-risk has also been reported in other settings [36].

The improvement of continuum of HIV services is a challenge everywhere. Interpersonal, intrapersonal, health system related and structural barriers are important impediments to goal achievement throughout the continuum [34]. However, three sets of mechanisms embedded in ten pathways were identified according to the interviews. Self-acceptance through self-esteem, awareness and pride, perception of HIV risk and acceptance and HIV status is a key component on addressing the intrapersonal barriers. The MSM can be more confident by focusing on greater self-awareness of emotions, goals, behaviors, and associated barriers while fostering acceptance of parts of the self that cannot change. Sense of community support by addressing community stigma, strengthening of MSM organizations and community networks allow MSM to help their peers not only for stigma reduction but also for engagement and retention in care. Sense of comprehensive and tailored HIV services with sexual stigma reduction training for healthcare providers, engagement of peer support and adapted services delivery through drug dispensing points and mobile technology contribute to better comprehensive care. Enhance motivation and adaptation by societal acceptation, tolerance and financial assistance contribute to better continuum outcomes by enhancing motivation and adaptation throughout the dynamism of the specific context.

Refining the IPT by mechanism identification, it appears that interventions are more effective when multiple layers of strategies are implemented together to address complex health programs, such as the continuum of HIV services. Thus, our refined program theory provides sufficient evidence to claim that multi-level pathways are essential for continuum outcomes improvement. With the expanding recognition of the importance of the continuum of HIV services for MSM on decreasing HIV morbidity, mortality and transmission, engagement, adherence and retention play an important on achieving a generation free of HIV by 2030. It is crucial that relevant mechanisms and pathways are included in every step on developing interventions for MSM.

A key limitation of this realist evaluation is that we were very restricted by including MSM who voluntarily disclosed their sexual orientation from only one healthcare setting due to data quality and consistency. Additionally, it is likely that some of the views shared during the interviews are based on perceptions and not lived experiences. As a result, we may have missed some other contextual factors, mechanisms and pathways. However, the LINKAGE intervention represented a pilot effort in order to expand the strategy to the entire network of HIV care throughout the country. Thus, we propose that the mechanisms and pathway outlined in this paper should be seen as the essential, rather than the only one contributing to better continuum outcomes. Another limitation is that we were not able to fully capture the specific components of each step of the continuum. In order to be able to focus on the general aspects, we have purposively overlooked these stratifications at a global level. Despite these limitations, the use of a realist evaluation is increasingly relevant according to the conceptualization of the intervention. The mixed methodology used added value in trying to achieve a complete view from triangulation and saturation. Our findings provide valuable insight into the inconsistency of the continuum and the relevant pathways and mechanisms. Additionally, to the best of our knowledge this is the first time that realist evaluation has been used to assess HIV complex interventions in Haiti as this approach is more suitable for exploring complex interventions.

## Conclusions

The study findings show that engagement, adherence and retention to the continuum of HIV service for MSM are affected by a multi-layer of factors, thus highlighting the importance of taking a comprehensive approach to improve the program.

## Data Availability

The materials, datasets used and analyzed during the current study are available from the corresponding author on reasonable request.

## Abbreviations

IPT: Initial program theory
HIV: Human immunodeficiency virus
HIV/AIDS: Human immunodeficiency virus and acquired immune deficiency syndrome
LTFU: Loss to follow-up
MSM: Men who have Sex with Men
PLHIV: People living with HIV
